# Intratumoral injection of holmium-166 microspheres as neoadjuvant therapy of soft tissue sarcomas in dogs

**DOI:** 10.3389/fvets.2022.1015248

**Published:** 2022-11-01

**Authors:** Nino Chiron Morsink, Johannes Frank Wilhelmus Nijsen, Guillaume Cornelis Maria Grinwis, Jan Willem Hesselink, Jolle Kirpensteijn, Sebastiaan Alexander van Nimwegen

**Affiliations:** ^1^Department of Clinical Sciences, Faculty of Veterinary Medicine, Utrecht University, Utrecht, Netherlands; ^2^Department of Medical Imaging, Radboud Institute for Health Sciences, Radboud University Medical Center, Nijmegen, Netherlands; ^3^Department of Biomolecular Health Sciences, Faculty of Veterinary Medicine, Utrecht University, Utrecht, Netherlands

**Keywords:** canine, holmium-166, intratumoral, microbrachytherapy, soft tissue sarcoma (STS)

## Abstract

**Introduction:**

Minimally invasive microbrachytherapy is in development to treat solid tumors by intratumoral injection of (radioactive) holmium-166 (^166^Ho) microspheres (MS). A high local dose can be administered with minimal damage to surrounding tissue because of the short soft tissue penetration depth of ^166^Ho beta radiation. We aimed to prospectively evaluate the safety and efficacy of ^166^Ho microbrachytherapy in client-owned canine patients with soft tissue sarcomas (STS).

**Methods:**

We included seven dogs with STS not suitable for local excision due to tumor size and/or location. ^166^HoMS were suspended in a carrier fluid and multiple needle-injections were performed in predetermined tumor segments to maximize tumor coverage. Tumor response was evaluated using 3D caliper and CT measurements. Follow-up further included monitoring for potential side effects and registration of subsequent treatments and survival, until at least two years after treatment.

**Results:**

Delivered radioactive doses ranged from 70 to 969 Gy resulting in a mean tumor volume reduction of 49.0 ± 21.3% after 33 ± 25 days. Treatment-related side effects consisted of local necrosis (*n* = 1) and ulceration of the skin covering the tumor (*n* = 1), which resolved with basic wound care, and surgical excision of residual tumor, respectively. Residual tumor was surgically resected in six patients after 22–93 days. After a mean follow-up of 1,005 days, four patients were alive, two patients were euthanized because of unrelated causes, and one patient was euthanized because of disease progression after the owner(s) declined subsequent surgical treatment.

**Conclusion:**

^166^Ho microbrachytherapy was a safe and effective neoadjuvant treatment option for canine patients with STS.

## Introduction

Soft tissue sarcomas (STS) are a heterogenous group of mesenchymal tumors with similar biological behavior but with distinct pathological characteristics (1–5). In the USA, STS is one of the five leading causes of cancer-related death in people under 39 years (6).

In dogs, STS are in the top five most common tumors, accounting for 8–15% of all skin and subcutaneous tumors, which is the most common site for tumor development (1, 5, 7). Over 50 histologic (sub)types have been identified in humans and animals (2, 3, 8). These diverse types are generally grouped based on the tissue or cell of origin, with the most common types in veterinary patients being: fibrosarcoma, pleomorphic sarcoma [previously named malignant fibrous histiocytoma (9)], hemangiopericytoma, and peripheral nerve sheath tumors (1–3, 5, 10).

The primary treatment of STS consists of wide surgical resection to achieve local tumor control, given that no metastases are found (5, 11, 12). Adjuvant external beam radiation therapy (EBRT) is often performed to prevent local recurrence resulting from contaminated surgical margins (5, 13). Reported five-year survival rate is 76% for dogs treated with curative intent EBRT after incomplete resection (14). Complete surgical resection often requires limb amputation because STS are most often found in body extremities (up to 60%), leaving these patients permanently impaired (7, 11, 13–16). Narrow excision has been effective for low-grade extremity STS but is only applicable in superficially located STS not showing invasive growth (4, 17, 18).

A new, minimally invasive treatment option for inoperable solid malignancies is currently being developed: intratumoral injection of (radioactive) holmium-166 (^166^Ho) microspheres (MS), named ^166^Ho microbrachytherapy (19–21). ^166^Ho is a promising radionuclide for microbrachytherapy because it emits high energy beta radiation (E_β,*max*_ = 1.85 MeV, t_1/2_ = 26.82 h) with a short soft tissue penetration depth (mean 2.2 mm, max. 8.7 mm), thereby enabling a high tumor dose with minimal risk for surrounding tissues (22–24). In addition, holmium is paramagnetic and has a high electron density, whereas ^166^Ho also emits gamma-rays (E_γ_ = 0.08 MeV, 6.6%), thereby enabling detection with magnetic resonance imaging (MRI), computed tomography (CT), and single-photon emission CT (SPECT), respectively.

In cats with oral squamous cell carcinoma (SCC), ^166^Ho microbrachytherapy induced a tumor volume reduction of 83 ± 22% with minimal side effects (20). CT-guided treatment of a Jack Russell Terrier with a pituitary macro-tumor resulted in 40% tumor volume reduction (25). ^166^Ho microbrachytherapy also proved to be a feasible treatment option in cats with liver tumors (26) and in human patients with head and neck SCC (27). In veterinary patients with STS, an intralesional brachytherapy using an injectable Yttrium-90 hydrogel has been evaluated, resulting in variable local responses (28).

The aim of this study was to prospectively evaluate the safety and efficacy of ^166^Ho microbrachytherapy in dogs with spontaneous STS.

## Methods

### Patient selection

Dogs with STS that were referred to the University Clinic for Companion Animal Health (Department of Clinical Sciences, Faculty of Veterinary Medicine, Utrecht University, Utrecht, Netherlands) between 2009 and 2013 were considered for inclusion. Inclusion criteria were a diagnosis of STS by histopathology, or a strong suspicion based on cytology together with findings from clinical examination and imaging studies, considered not suitable for wide surgical resection due to either its size, location, infiltrative growth, or a combination. Additional inclusion criteria were the absence of detectable metastases and severe comorbidities such as renal or liver failure. Exclusion criteria were the dog receiving chemotherapy or other specific anti-tumor therapies, or surgery within four weeks prior to study entry.

### Diagnosis and staging

Patient data were recorded including breed, sex, age, weight, and clinical history. Each patient underwent general and physical examination, including tumor inspection, palpation, and tumor size evaluation by 3D caliper measurements. Blood and urine were analyzed to screen for other (sub)clinical disorders.

Guidelines for staging STS in dogs were followed (4, 5, 29), including (contrast-enhanced) CT (Supplementary Table 1; Secura, Philips Medical Systems, Best, Netherlands; 1.3–2.2 ml/kg, Xenetix 350 mg/ml, Guerbet, Villepinte, France), to further assess tumor size, invasion of surrounding tissues, and possible metastases (30). In case of abnormal regional lymph nodes, ultrasound-guided fine needle aspiration biopsy (FNAB) was performed with subsequent cytologic examination. FNAB and cytology of the tumor was included if a histopathologic diagnosis was not available.

For each patient, tumor volume was calculated assuming ellipsoid shape (Equation 1) using the three longest perpendicular diameters as measured manually and on CT.


(1) $$\begin{array}{l} Volume=\frac{\pi }{6}\times length\times width\times height \end{array}$$

### Radionuclide treatment facility

Patients were treated in the radionuclide treatment facility of the University Clinic for Companion Animal Health, consisting of a treatment room, hospitalization wards, a radionuclide lab, and a SPECT room. After ^166^Ho microbrachytherapy, SPECT was performed, and patients were hospitalized in our radionuclide wards.

### Holmium microspheres and syringe preparation

Holmium-165 acetylacetonate microspheres (^165^HoAcAcMS) and holmium-165 poly-L-lactic acid microspheres (^165^HoPLLAMS) were produced by our research group as previously described (31, 32). ^165^HoMS were neutron irradiated at the Reactor Institute Delft (Delft University of Technology, Delft, Netherlands) to obtain the predetermined specific radioactivity (MBq/mg ^166^HoMS) for each patient. The ^166^HoMS were suspended in sterile water containing 2% poloxamer 188 (Pluronic F-68, Sigma-Aldrich Chemie, Zwijndrecht, Netherlands) by gentle agitation and repeatedly drawing up and down in a syringe. Aliquots of 0.4 ml were drawn up into separate 1 ml Luer-lock syringes (Plastipak, Becton Dickinson, Vianen, Netherlands). Multiple syringes were prepared for each patient, based on tumor volume and consistency. The amount of radioactivity in each syringe was measured in a dose calibrator (VDC-404, Comecer, Joure, Netherlands). To limit exposure of personnel to beta radiation, each syringe was placed into an 8-mm thick acrylic glass cylinder during preparation and treatment.

### Dose calculation

We aimed for a mean tumor-absorbed dose of at least 200 Gy (J/kg), equal to the low dose protocol in the intratumoral treatment of feline oral SCC (20). The required ^166^Ho radioactivity was calculated using Equation 2 derived from the medical internal radiation dosimetry pamphlet number 17 (33), as widely used for Yttrium-90 dosimetry (34), and more recently for ^166^Ho dosimetry in liver malignancies (35) and intratumoral applications (20, 25, 27). We calculated the required radioactivity for a mean tumor dose of 200 Gy assuming homogenous ^166^HoMS distribution in the tumor. We prepared the syringes with an added 50% of radioactivity to prevent underdosing because the mean injection efficiency was 60% in a previous study due to ^166^HoMS sedimentation in the injection system (20).


(2) $$\begin{array}{l} A=\frac{D\;\times \;W}{15.87} \end{array}$$

A = ^166^Ho radioactivity (MBq); D = tumor-absorbed dose (Gy); W = tumor weight (g), assuming a tumor tissue density of 1.06 g/cm^3^ based on Report 44 of the International Commission on Radiation Units and Measurements (36); ^166^Ho-specific tissue dose conversion coefficient = 15.87 mJ/MBq (36–38) based on S-values as previously calculated using Monte-Carlo simulations (37).

### Anesthesia, analgesia, medication

^166^Ho microbrachytherapy was performed under general anesthesia with endotracheal intubation. Premedication included intravenous (IV) administration of dexmedetomidine hydrochloride (0.01–0.02 mg/kg, Dexdomitor, Zoetis, Capelle aan de IJssel, Netherlands), or midazolam (0.30 mg/kg, Veterinary Medicine Pharmacy, Utrecht, Netherlands) and butorphanol (0.30 mg/kg IV, Dolorex, Intervet, Boxmeer, Netherlands).

General anesthesia was induced by propofol (1–2 mg/kg IV, Propovet, AST Farma, Oudewater, Netherlands) and maintained by inhalation of isoflurane (1.5–2.5%, IsoFlo, Abbott Animal Health, Illinois, USA) in O_2_/air (1:1). In patient 5 with cardiac disease, alfaxalone was used for induction (1 mg/kg IV, Alfaxan, Jurox, West Sussex, United Kingdom). Anesthesia monitoring consisted of heart rate, respiratory rate, body temperature, non-invasive blood pressure, pulse oximetry, capnography, and end-expiratory isoflurane partial pressure measurement. Patients were recovered by intramuscular administration of atipamezole hydrochloride (0.05–0.10 mg/kg, Antisedan, Zoetis), except patient 5.

Analgesia included either or both buprenorphine (0.01–0.02 mg/kg IV, Buprecare, AST Farma) and carprofen (4 mg/kg IV, Rimadyl, Pfizer, Capelle aan de IJssel, Netherlands).

Post-operative medication included oral administration of carprofen (2 mg/kg, twice daily for 5–7 days, Carporal, AST Farma) and tramadol hydrochloride (2–3 mg/kg, 3–4 times daily for 5–10 days, Pharmacy Veterinary Medicine).

### Holmium-166 microsphere treatment

Patients were prepared by clipping and anti-septic preparation of the skin. The floor, working surfaces, and treatment table were covered with disposable absorbent foil to prevent radioactive contamination. Patients were positioned on the treatment table and covered with surgical draping. The tumor area was divided in visually equal tumor segments using a sterile skin marker, representing part of the tumor volume, according to the treatment plan. We aimed to inject the radioactive suspension of at least one syringe per tumor segment in all patients. We performed multiple ^166^HoMS injections symmetrically in these segments at various depths, aiming for a maximum distance of 6 mm between depots and to the tumor margin for optimal tumor coverage. Syringes were rotated horizontally to suspend sedimented ^166^HoMS before each injection. Approximately 4 depots of 0.1 ml were injected per syringe. To increase injection efficiency, the visible ^166^HoMS residue in the syringe and needle after injection was resuspended once or twice per syringe by aspiration of ~0.2 ml of sterile 0.9% NaCl solution and rotated again, after which injections were continued.

We routinely used 22G needles (Spinocan, B. Braun, Melsungen, Germany) of various lengths depending on tumor size. However, based on earlier experience (20), 24−27G needles (Sterican, B. Braun) were used for tumors with smallest diameter or firm consistency to prevent backflow through needle tracks. Gauze sponges were placed against the injection site after needle retraction to collect potential leakage.

After treatment, the tumor site was cleaned repeatedly using moist gauze sponges to detect and remove possible radioactivity that leaked out of needle tracts. The gauze sponges were immediately measured for radioactivity. If radioactivity was found in gauzes after wiping the treatment area, cleaning was continued until measured radioactivity levels were negligible. A temporary gauze bandage was taped over the treatment area to prevent possible ongoing leakage and risk of spread of ^166^HoMS during SPECT imaging and recovery of the patient. An Elizabethan collar was placed in most patients during recovery. The collar was often removed the next morning since most dogs were not interested in the treated area. Radioactivity in the syringes, needles, and disposables (e.g., gauze sponges and gloves) were measured in the dose calibrator. The amount of injected radioactivity was calculated by subtracting the post-treatment measurements from the pre-treatment measurements, after correcting the data for radioactive decay until time of treatment (24).

### Post-operative imaging

We assessed local ^166^HoMS deposition in the tumor and possible unintended spread immediately after treatment using anterior-posterior and lateral planar gamma scintigraphy (Orbiter 37, Siemens Medical Systems, Illinois, USA; SKYLight, Philips Medical Systems). A medium-energy general-purpose collimator was used with energy windows set to 80.6 keV ± 7.5% for the ^166^Ho photopeak and 118.0 keV ± 6.0% for correction for down-scattered high-energy photons, as previously described (38).

### Post-operative care

Patients were monitored daily, including general and physical examination, tumor inspection, and blood and urine analysis if indicated. An Elizabethan collar was (re)placed in case of frequent licking of the tumor site and feces were collected and measured for radioactivity. Patients were discharged when the external dose rate was below the local regulatory limit of 1 μSv/h at 1 m distance, as measured using a dose rate meter (RDS-100, Alnor, Minnesota, USA). The owner(s) received radiation safety instructions for the care of the dog in the first week after discharge.

### Follow-up

The standard follow-up protocol consisted of hospital visits after two weeks, four weeks, three months, and six months. Follow-up consisted of general and physical examination, including tumor evaluation, and additional laboratory investigation or diagnostic imaging if indicated based on clinical signs or local disease progression.

After six months, patients were monitored through regular contact with the owner(s) for at least two years after treatment. Recorded long-term follow-up data included potential side effects, disease recurrence or metastases, subsequent treatments, and survival.

### Tumor response

We evaluated post-treatment tumor size by 3D measurements using a caliper or using CT if the residual tumor could not be measured accurately by hand. Tumor response was scored as percentage tumor volume change between pre- and the post-treatment tumor volumes (Equation 1) that resulted both from caliper measurements and both from CT measurements. For each patient, we selected the post-treatment volume that showed maximum response.

### Subsequent treatments

Subsequent treatments were considered based on treatment response, clinical feasibility and perceived prognosis, and the wish of the owner(s), and could include a second ^166^HoMS treatment, surgical excision, and EBRT.

### Histopathologic examination

In case of surgery following the ^166^HoMS treatment, histopathologic examination was performed of the excised tumor tissue to determine tumor type and grade, and to estimate the amount of inflammation, necrosis, and ^166^HoMS present (none-minimal-moderate-high).

### Long-term outcome

To quantify the long-term outcome, we calculated the overall survival (OS), defined as the time from ^166^HoMS treatment until death, and the disease-free survival (DFS), defined as the time from ^166^HoMS treatment until the first signs of local recurrence or metastases.

### Statistical analysis

Numerical data are presented as the mean ± standard deviation if normally distributed and as the median and interquartile range (IQR: Quartiles 1–3) if skewed based on the Shapiro-Wilk test with *p* ≤ 0.05. Categorical data are presented as numbers and percentages. Statistical analysis was conducted using IBM SPSS Statistics 27.

## Results

### Patients

We included seven client-owned dogs (two males, five females) aged 9.2 ± 1.8 years and weighing 30.9 ± 13.9 kg (Table 1). Tumors were located subcutaneously around the radius/ulna (*n* = 2), the tarsus (*n* = 1), the femur (*n* = 3), and the elbow (*n* = 1). Patient 1 was referred with local recurrence after the tumor had been surgically excised twice by the referring veterinarian. Patient 6 was referred with a large, compartmentalized cystic tumor, which was drained twice before referral. The other patients were referred without prior treatment of their STS.

**Table 1:** Patient characteristics and preparation of holmium-166 microbrachytherapy of seven canine patients with soft tissue sarcoma.

**Patient no**.	**Patient characteristics**				**Diagnosis and staging**										**HoMS and syringe preparation**				
	**Breed**	**Sex**	**Age (years)**	**Weight (kg)**	**Tumor location**	**Largest diameter (cm)**	**Tumor volume (cm^3^)**	**Time pre-treatment (days)**	**Consistency **	**Movable**	**Well-defined on palpation**	**Well-defined on CT**	**TNM stage **	**Cancer stage**	**HoMS type**	**HoMS (mg)**	**Holmium (%)**	**No. of syringes**	**Syringes (MBq)**
1	DS	F	8.0	22.6	Right dorsal radius	5.2	40.7	14	Firm	No	Yes	No	T2bN0M0	I	PLLA	153	18.5	6	1,054
2	SI	F	7.2	7.2	Left dorsal tarsus	4.1	16.2	7	Soft	No	Yes	No	T1bN0M0	I	NA	NA	NA	NA	NA
3	CB	F	8.0	49.5	Left proximal femur	7.0	110.0	7	Firm	No	Poor	No	T2aN0M0	I	PLLA	740	18.5	12	2,093
4	CB P	M	10.0	37.9	Left lateral radius / ulna	5.7	52.2	0	Fluctuating / soft	No	Yes	No / moderately	T2bN0M0	I	AcAc	293	43.0	11	2,544
5	IS	F	12.7	28.5	Left proximal femur	8.9	165.1	35	Firm	No	Yes	NA	T2bN0M0	I	AcAc	199	43.0	12	2,765
6	CB	F	9.1	42.2	Left proximal femur	9.0	261.3^*^	35	Fluctuating with firm contents	No	Moderately	No / moderately	T2bN0M0	I	AcAc	397	43.0	12	2,739
7	ST	M	9.7	28.7	Right elbow	11.7	372.5	22	Soft / irregular	No	Moderately	No / moderately	T2bN0M0	I	PLLA	222	15.0	7	2,155

Guidelines were followed for staging STS in dogs **(****4**, **5**, **29****)**. DS, Dutch shepherd; SI, Shiba Inu; C, crossbreed; P, poodle; IS, Irish setter; ST, Staffordshire terrier; F, female; M, male; CT, computed tomography; PLLA, poly-L-lactic acid; AcAc, acetylacetonate; NA, not available.

* Tumor volume calculated using CT measurements instead of caliper measurements. Tumor volumes were calculated using Equation 1.

### Diagnosis and staging

In all patients, examination revealed no life-threatening comorbidities. All tumors were fixed to deeper tissues (fascia, muscles) on palpation (Table 1). Patient 5 was also diagnosed with supraventricular tachycardia but was deemed healthy enough for ^166^Ho microbrachytherapy, albeit by minimizing depth, duration, and number of anesthesia events.

**Figure 1:**
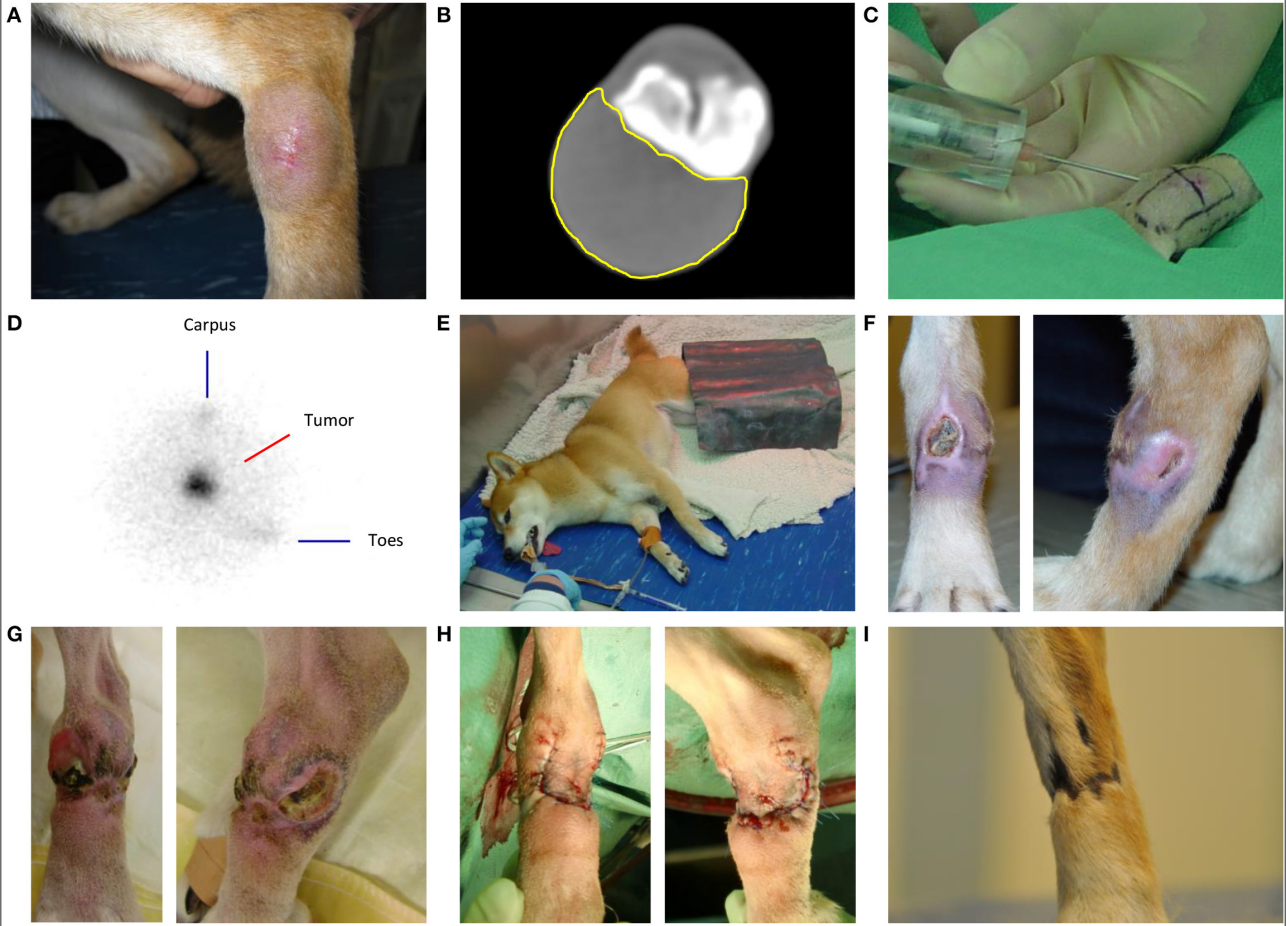
Intratumoral holmium-166 microsphere (^166^HoMS) treatment and follow-up of patient 2 with a soft tissue sarcoma in the left dorsal tarsus. **(A)** The tumor seven days before treatment. **(B)** Computed Tomography of the tumor five days before treatment, showing the transverse slice with the largest surface area of the tumor (yellow line). **(C)** Needle-injection of ^166^HoMS in four predetermined and marked tumor segments. An 8-mm thick acrylic glass cylinder was placed around the syringe to limit unwanted beta radiation exposure of personnel and the patient. **(D)** Right lateral planar gamma scintigraphy image of the tumor after treatment, showing concentrated gamma counts in the tumor area (center). **(E)** Recovery of the patient in our dedicated radionuclide ward. A lead plate was placed over the tumor to limit gamma radiation exposure of personnel. **(F)** The tumor 29 days after treatment, showing a medial ulceration of ~1.0 × 1.0 cm and a lateral ulceration of ~1.0 × 0.5 cm. **(G)** The tumor 93 days after ^166^HoMS treatment, showing multiple ulcerations, inflammation, and distal edema formation because of constant licking. **(H)** The patient's leg after surgical resection of the tumor, 93 days after ^166^HoMS treatment. **(I)** The patient's leg 281 days after ^166^HoMS treatment and 188 days after surgical resection, showing dark and hairless scar tissue at the location of the resected tumor.

**Figure 2:**
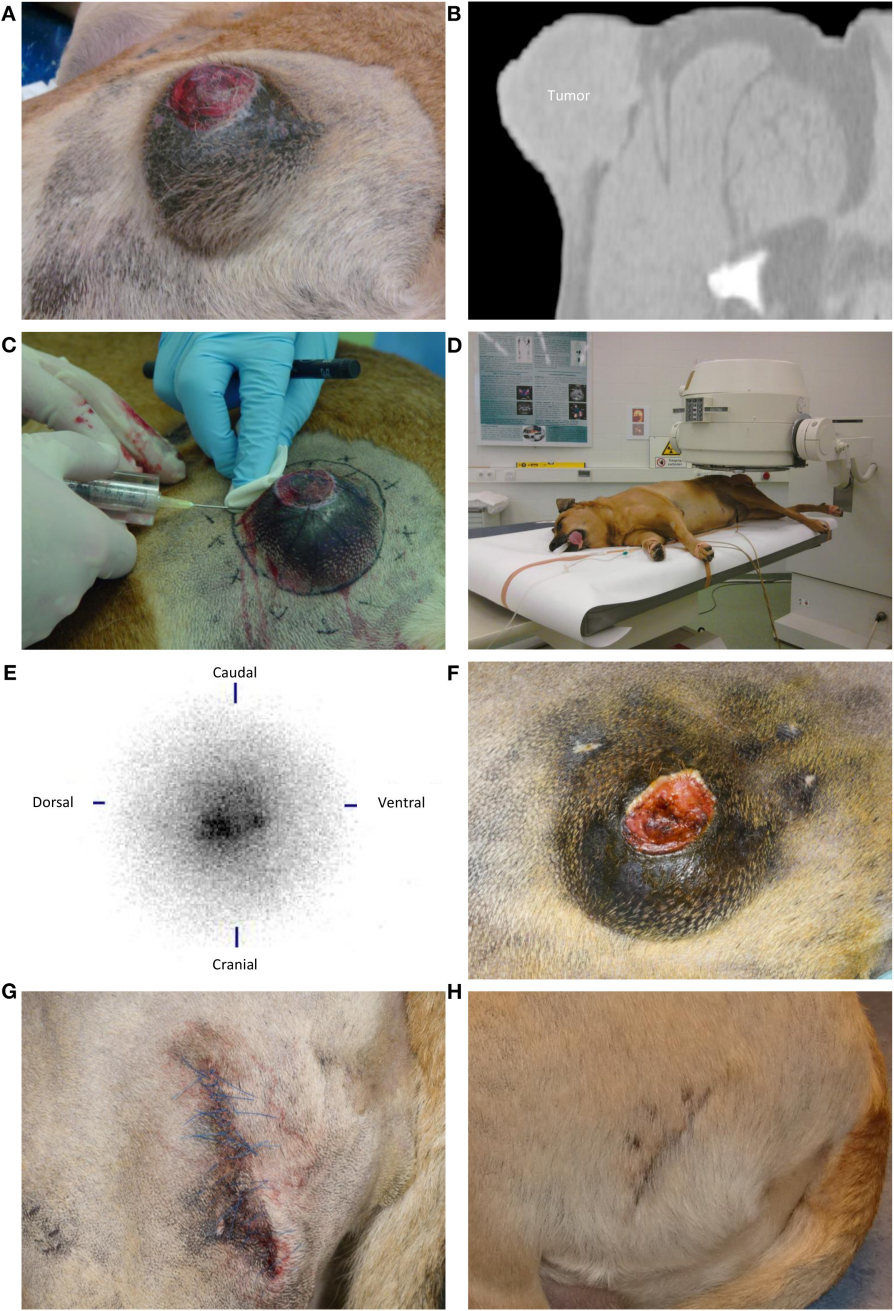
Intratumoral holmium-166 microsphere (^166^HoMS) treatment and follow-up of patient 3 with a soft tissue sarcoma in the left proximal femur. **(A)** The tumor seven days before treatment, showing an ulcerative lesion of 1.5 × 1.5 cm in the center of the tumor. **(B)** Computed Tomography of the tumor seven days before treatment, showing a transverse slice with evident infiltration of the tumor into subcutaneous tissues. **(C)** Needle-injection of ^166^HoMS in 12 predetermined and marked tumor segments. An 8-mm thick acrylic glass cylinder was placed around the syringe to limit beta radiation exposure of personnel and the patient. **(D)** Left lateral planar gamma scintigraphy of the tumor after treatment. **(E)** Left lateral planar gamma scintigraphy image of the tumor after treatment, showing concentrated gamma counts in the tumor area (center). **(F)** The tumor 36 days after treatment, showing an 82% decrease in tumor volume compared to Figure 2A, with still an ulcerative lesion in the tumor center. **(G)** The patient's hip after surgical resection of the tumor, 36 days after ^166^HoMS treatment. **(H)** The patient's hip 63 days after ^166^HoMS treatment and 27 days after surgical resection.

The tumor volumes before treatment ranged from 16.2 to 372.5 cm^3^ (Table 1, Supplementary Table 2). In six patients, the tumor did not result in overt clinical signs (Figure 1A). Patient 3 had an ulcerative lesion of 1.5 × 1.5 cm of the skin covering the tumor (Figure 2A).

CT revealed that all tumors showed either local infiltrative growth in surrounding tissues or loss of detail in the deep margin (Table 1, Figure 1B). In patient 3, infiltration of the tumor through the pseudo-capsule into subcutaneous tissues was evident (Figure 2B). In patients 1, 4, and 7 tumor infiltration into or between adjacent muscles was apparent. In patients 2 and 6 infiltration could not be ruled out. Five patients had no signs of metastasis. Patient 3 had a single 7 mm diameter focal, poorly defined soft tissue opacity in the accessory lung lobe for which metastasis could not be completely ruled out but was considered unlikely. Patient 7 had a slightly enlarged regional lymph node, without evidence of metastasis on cytology. Patient 5 with cardiac disease was not evaluated with CT, but thoracic x-rays showed no signs of metastasis.

All patients received a tentative diagnosis of spindle cell sarcoma: two patients (1 and 3) based on histopathologic examination without immunohistochemical staining after previous surgical excision, and five patients based on cytologic examination.

### Holmium microspheres and syringe preparation

The total radioactivity in the syringes before treatment of six patients ranged from 1,054 – 2,765 MBq divided over 6–12 syringes (Table 1, Supplementary Table 3). The radioactivity data of patient 2 were lost after calculation of the injected radioactivity.

### Holmium-166 microsphere treatment

The injected radioactivity ranged from 845–2,645 MBq, resulting in a tumor dose of 369 ± 320 Gy (range 70–969 Gy) (Table 2). The tumors were divided in up to 15 segments for ^166^HoMS injections (Figures 1C, 2C). Injection efficiency was 85 ± 6% in six patients, excluding missing data from patient 2. Radioactivity in the disposables of patients 1 and 3 was not measured or was lost and could not be included in these calculations.

**Table 2:** Results of holmium-166 microbrachytherapy of seven canine patients with soft tissue sarcoma.

**Patient no**.	**HoMS treatment**							**Tumor response**			**Follow-up**		**Long-term outcome**			
	**Anesthesia (h:min)**	**No. of tumor segments**	**Syringes (MBq)**	**Disposables (MBq)**	**Injected**		**Mean dose (Gy)**	**Tumor volume (cm^3^)**	**Time post-treatment (days)**	**Tumor response**	**Side effects**	**Subsequent treatment(s)**	**Final follow-up or death (days)**	**DFS (days)**	**OS (days)**	**Alive or cause of death**
					**(MBq)**											
															
															
															
1	01:53	1	209	NA	845	80%	311	27.8	5	−32%	Skin necrosis	Sx 85 days, Amp 223 days	1,637	159		Alive with amputated leg
2	01:21	4	NA	NA	1,048	NA	969	5.8	29	−64%	Tumor ulceration	Sx 93 days	1,959	1,947		Alive with late recurrence
3	01:20	12	400	NA	1,693	81%	321	19.8	36	−82%	Delayed wound healing^**^	Sx 36 days	755		755	Euthanasia due to hemoabdomen
4	01:54	8	252	72	2,220	89%	636	24.3	84	−54%		None	410		410	Euthanasia due to progression
5	01:14	8	320	90	2,355	85%	214	77.0	21	−53%		Sx 22 days	275		275	Euthanasia due to unrelated disease
6	02:00	8	87	7	2,645	97%	152	153.1^*^	37	−41%	Fluid accumulation^**^	Sx 36 days	840			Alive
7	01:42	15	344	75	1,736	81%	70	307.8	20	−17%		Sx 40 days, Sx 306 days, RTx 316 days	1,160	276		Alive

NA, not available; Gy, Gray (J/kg); Sx, surgical resection; Amp, surgical leg amputation; RTx, radiation therapy; DFS, disease-free survival: Time from holmium-166 microsphere (^166^HoMS) treatment until first clinical signs of recurrence including metastases; OS, overall survival: Time from ^166^HoMS treatment until death.

* Tumor volume calculated using CT measurements instead of caliper measurements. Tumor volumes were calculated using Equation 1.

** Side effect resulting from pre-existing clinical condition that continued to exist after treatment.

Prior to treatment of patient 6, ~300 ml of fluid was drained from the cystic part of the tumor.

### Post-operative imaging

Post-treatment SPECT confirmed local ^166^Ho deposition without unintended spread to surrounding tissues in patients 1–6 (Figures 1D, 2D,E). SPECT was not available during treatment of patient 7.

### Post-operative care

All patients recovered uneventful (Figure 1E) and were discharged after five to eight days without observed side effects or clinical decline.

### Tumor response

The median tumor volume changed from 110.0 cm^3^ (IQR 40.7–261.3 cm^3^) before treatment to 27.8 cm^3^ (IQR 19.8–153.1 cm^3^) after treatment (Table 2, Supplementary Table 2). The mean tumor volume reduction was 49.0 ± 21.3% after 33 ± 25 days.

The tumor volume reduction was accompanied by a softer tumor consistency in two patients (4 and 7) after three weeks.

### Side effects

Two patients (1 and 2) developed side effects at the injection site during follow-up (Table 2). Patient 1 presented with necrosis of the skin covering the tumor after 20 days, which healed within one month with basic wound care. Patient 2 presented with two deep ulcerative skin lesions after 29 days, located medial and lateral in the tumor area measuring ~1.0 × 1.0 cm and ~1.0 × 0.5 cm, respectively (Figure 1F). An open, inflamed wound was observed after 55 days, frequently licked by the dog despite wearing an Elizabethan collar and treatment with wound ointment. The tumor area was red, inflamed, and painful with ulcerative lesions measuring 6–15 mm in diameter and distal edema formation as observed after 93 days, right before surgical resection (Figure 1G).

Two patients (3 and 6) continued to suffer from their pre-existing clinical condition after treatment. Patient 3 with a pre-existing ulcerative wound also managed to continue licking it after treatment and suffered incomplete, delayed wound healing as observed after 36 days, right before surgical resection (Figure 2F). Patient 6 presented again with fluid accumulation in the cystic part of the tumor after 22 days. Surgical resection was performed two weeks later.

### Subsequent treatments

In all patients, tumor volume reduction facilitated subsequent surgical resection with narrow margins, which was performed in six patients after 22–93 days (Table 2, Figures 1H,I, 2G,H). In patient 4, tumor volume reduction was 54% after 84 days and surgical resection was advised but declined by the owner(s).

Tumor excision was marginal in all cases and surrounding skin was spared as much as possible to enable direct closure. In two patients (3 and 6), the tumor seemed to be completely removed, albeit with narrow margins on histopathology. In the other four patients (1, 2, 5, and 7), tumor resection was incomplete based on macroscopic and histopathological assessment. During surgery of patient 7, complete narrow surgical resection was not deemed possible because of tumor branches invading surrounding tissue including fascia and muscle, and rupture of the pseudo-capsule occurred twice. This necessitated dissection on the tumor edge/debulking of these tumor parts. The attempted marginal excision also caused significant blood loss because of the highly vascularized tumor surroundings/pseudo-capsule.

After marginal surgical resection, wound complications occurred in varying degrees in five out of six patients. In two patients (2 and 6), the operation wound was closed under tension which resulted in limited central wound dehiscence that healed relatively quickly by secondary intention with conservative wound care. In one patient (5), chronic superficial inflammation of the skin was observed, which healed slowly (three months) because of frequent licking, despite the advice to wear a collar. In two patients (1 and 7), chronic deep wound infection was observed, which was treated conservatively by wearing a collar, wound bandage, and ointment. This caused delayed wound healing in patient 7 because of a small chronic draining tract for two months. The wound complication was resolved after 4.5 months by surgical amputation in patient 1 because of concomitant local tumor recurrence.

Three patients (1, 2, and 7) with incomplete resected tumors on histopathology developed local recurrence after ^166^HoMS treatment and subsequent surgical resection. In patient 1, recurrence was observed 74 days after resection and limb amputation was performed 223 days after ^166^HoMS treatment. In patient 2, recurrence was observed 1,854 days after resection, and further treatment was advised but declined by the owner(s). In patient 7, recurrence was observed 236 days after resection. Marginal excision was performed for a second time 306 days after ^166^HoMS treatment, followed by EBRT (5 × 10 Gy) 10 days later because of incomplete margins and a high tumor grade on histopathologic analysis.

### Histopathologic findings

**Figure 3:**
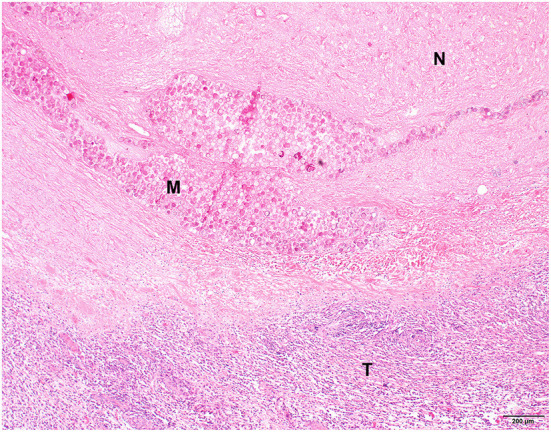
Histopathological picture of a spindle cell sarcoma of patient 3, 36 days after holmium-166 microspheres injection. Extensive necrosis is visible in the upper part of the image (N) with several foci containing microspheres (M). The bottom part of the image shows remaining neoplastic tissue with moderate lymphoplasmacytic inflammation (T). Hematoxylin and eosin stain.

For six patients, we performed histopathologic examination of resected tumor tissue without immunohistochemical staining and confirmed the presence of a spindle cell sarcoma (Table 3, Figure 3). Five patients had low-grade tumors and one patient (7) initially had a low-grade tumor but developed a high-grade recurrent tumor. The amount of tumor necrosis varied between patients and between initial tumor and recurrence in patient 1, but not in patient 7. We observed a varying degree of inflammation in the tumors, mainly lymphoplasmacytic and histiocytic. In samples from three patients (1, 3, and 4), we evidently found foci containing ^166^HoMS which were mainly located in necrotic tissue.

**Table 3:** Histopathologic findings of resected tumor tissue following holmium-166 (^166^Ho) microbrachytherapy of seven canine patients with soft tissue sarcoma.

**Patient no**.	**Time since ^166^Ho**	**Subsequent**	**Tumor grade**	**Necrosis**	**Microspheres**
	**treatment (days)**	**treatment(s)**			
1	85	Sx	Low	+++	+++
	223 (recurrence)	Amp	Low	–	–
2	93	Sx	Low	++	+++
3	36	Sx	Low	++	++
4	NA
5	22	Sx	Low	+++	–
6	36	Sx	Low	–	–
7	40	Sx	Low	+	–
	306 (recurrence)	Sx	High	+	–

Histopathologic examination was performed without immunohistochemical staining and confirmed spindle cell sarcoma in all patients. An illustration of necrosis and microspheres present in a tissue sample is shown in Figure 3. Sx, surgical resection; Amp, surgical leg amputation.

### Long-term outcome

Follow-up duration was 1,005 ± 621 days (Table 2). Four patients (1, 2, 6, and 7) were alive at final follow-up. Patient 1 was alive after 1,637 days with an amputated leg. Patient 2 was alive after 1,959 days with local recurrence discovered 12 days earlier. Patient 6 was alive after 840 days, despite the discovery of an inoperable and metastasized anal sac carcinoma after 750 days. Patient 7 was alive and disease free after 1,160 days following ^166^HoMS treatment, surgical excision (twice), and EBRT. In all patients, we did not find indications for metastases related to the initial tumor.

Two patients (3 and 5) were euthanized presumably for causes unrelated to the tumor. Patient 3 collapsed and was euthanized after 755 days due to hemoabdomen of unknown origin without previous signs of clinical decline or tumor recurrence. Patient 5 was euthanized after 275 days, after being admitted to our intensive care unit with severe regenerative anemia without apparent signs of blood loss and no signs of metastases on thoracic x-rays and abdominal ultrasound. Blood transfusions shortly improved the clinical situation, but the anemia returned despite corticosteroid therapy. Further diagnostics and treatment were discontinued as desired by the owner(s).

Patient 4 was euthanized after 410 days by the referring veterinarian because of local disease progression and at request of the owner(s).

## Discussion

To our knowledge, we were the first to treat canine patients with STS by intratumoral injection of radioactive microspheres. We treated seven dogs by ^166^Ho microbrachytherapy, delivering tumor doses of 70–969 Gy resulting in a tumor volume reduction of 49.0 ± 21.3% without severe periprocedural side effects. After a mean follow-up of 1,005 days, four patients were alive, two patients were euthanized because of unrelated causes, and one patient was euthanized because of local disease progression.

After ^166^Ho microbrachytherapy-inflicted tumor volume reduction, marginal surgical excision was performed in all but one patient (4), because the owner(s) declined further treatment. OS of patient 4 of 410 days might have been longer if subsequent surgery was performed. Patient 2 was alive at 1,959 days but was not treated for a late recurrence due to costs. Unfortunately, treatment may not always be pursued depending on the wishes and financial situation of the owner(s).

After marginal surgical resection, wound complications occurred in varying degrees in five out of six patients. In most patients, dissection was very close under the skin edges to spare skin for primary wound closure, which was still under relative tension in several cases. Wound dehiscence is not uncommon under these circumstances, but would normally resolve by conventional wound treatment, as observed in patients 2 and 6. In patient 5, only minor wound inflammation was observed after surgical resection, but local irritation and chronic thickening of the skin developed, possibly because of frequent licking (lick granuloma). However, we cannot rule out the possibility of local radiation trauma from the ^166^HoMS treatment causing irritation and delayed wound healing. Likewise, radiation damage may have contributed to delayed wound healing and chronic wound infection in patients 1 and 7. Wound complications did not affect long-term prognosis.

Histopathological assessment after ^166^Ho microbrachytherapy was somewhat complicated because there were no pre-treatment samples available, and necrosis and inflammation could have been present in the initial tumor. Furthermore, the timeframe of histopathological changes after ^166^Ho microbrachytherapy in STS is unknown and the time until subsequent surgical excision varied between patients. However, ^166^HoMS were mainly found in necrotic tumor parts and the high necrosis score in the tumor of patient 1 after ^166^Ho microbrachytherapy was not apparent in the local recurrence that developed later, supporting that ^166^Ho microbrachytherapy induced tumor necrosis as previously described (21, 37, 39). It is, however, not possible to evidently relate the observed necrosis and inflammation to the ^166^Ho microbrachytherapy in this clinical study.

Patient 7 developed a high-grade local recurrence after excision of a low-grade STS after ^166^Ho microbrachytherapy. It is unknown if this progression in tumor grade may be linked to the ^166^Ho microbrachytherapy or that the marginal excision left skip-lesions of higher malignancy. Information on progression of malignant features in local recurrent sarcomas is very sparse in humans (40) and not available for dogs.

Two patients with the smallest tumors developed side effects presumably related to the ^166^HoMS treatment: Patient 1 had skin necrosis and patient 2 had deep ulcerative lesions. This could indicate a higher risk of backflow of the ^166^HoMS which could lead to a high dose of the skin or subcutaneous tissue, possibly because of the shorter injection canals and relatively larger injection volumes. However, patient 2 also had the highest tumor dose, 969 Gy in 16.2 cm^3^, which may also have induced a relatively high dose to the skin covering the tumor. These side effects may be reduced or prevented by reducing the number and volume of injections and restricting injections to the tumor center. However, spatial ^166^HoMS distribution in the whole tumor is required to achieve complete dose-coverage, as previously described (20, 25, 27).

On the contrary, the mean doses were lower than aimed for (200 Gy) in patients 6 and 7 with the largest tumors, 152 Gy in 261.3 cm^3^ and 70 Gy in 372.5 cm^3^, respectively. Patient 7 also showed the smallest tumor volume reduction (−17%), which may be the result of the lower dose and possible inhomogeneous distribution of ^166^HoMS. Covering the tumor completely is especially challenging in larger tumors as a larger distribution of ^166^HoMS is needed, which depends on tumor consistency and the number and locations of the injections. Additionally, larger tumors require more total radioactivity, and we reached the regulatory limit ( ≤ 2.5 GBq) in the treatment preparation of patients 5–7. Maybe we could have improved ^166^HoMS distribution in these tumors by dividing the ^166^HoMS over more injections, but this comes with a higher risk of tissue damage, leakage at the cutaneous injection site, peritumoral accumulation of ^166^HoMS, and radiation exposure of personnel. However, there is limited data on the administration of high intratumoral doses and the resulting tumor response. Future studies should focus on this relationship while factoring in different injection strategies in relation to tumor size.

We expect to improve safety and efficacy of ^166^Ho microbrachytherapy by implementing quantitative imaging. MRI is currently being used after radioembolization of human patients for assessment of ^166^HoMS biodistribution and dosimetry (41–44). Recently, CT has been used to confirm proper needle positioning prior to ^166^HoMS injections (25) and for ^166^HoMS quantification (45). MRI or CT guidance could have been of great added value in the present study to monitor ^166^HoMS distribution intraoperatively. However, CT- or MRI-guided ^166^Ho microbrachytherapy are currently under development within our research group (45) and were not available at the time of the study. At last, combined SPECT-CT imaging would be essential in future studies to provide anatomical reference to the detected radioactivity after treatment.

The efficiency of ^166^HoMS delivery from the syringe into the patient of 80–97% was markedly higher than 50–60% delivery reported in previous intratumoral ^166^HoMS studies (20, 21). One important difference is that we resuspended sedimented ^166^HoMS after emptying a syringe and injected again, which we recommend for future ^166^HoMS studies that aim to treat solid tumors suitable for multiple needle injections.

We did not categorize tumor response according to World Health Organization criteria (30, 46, 47), Response Evaluation Criteria in Solid Tumors (48, 49), or previously published volumetric criteria (50) because these methods assume spherically shaped tumors and uniform tumor size changes based on unidirectional and bidirectional measurements. We measured tumor size in 3D (length, width, and height), which also has been used extensively for assessment of tumor response, including in previous ^166^Ho microbrachytherapy (20, 25, 51, 52). We used caliper measurements in most patients to calculate tumor response, whereas we used CT in only one patient (6). It would have been more accurate to use CT in all patients for comparative response evaluation (53). For future studies, we recommend acquiring at least one CT dataset after treatment to measure tumor size and monitor possible unexpected and otherwise undetected side effects, although this requires additional anesthesia for the patient and more time and costs for the owner(s).

Some data were missing in our study results. For patient 2, the forms with measured radioactivity were lost, but the resulting injected radioactivity value was available which is most important. For patients 1 and 3, measurements of the disposables were not available, which may have led to an overestimated tumor dose. However, this overestimation is assumed to be minimal since the disposables of other patients only contained up to 3% of total radioactivity. For patient 5 with cardiac disease, CT was not acquired to reduce anesthesia events. For patient 7, SPECT was unavailable after treatment due to maintenance issues. At last, STS subtype was not confirmed in six patients because immunohistochemical staining was not performed. STS subtype and grade could not be confirmed in patient 4 because surgery was not performed.

## Conclusion

The results of this study demonstrate that ^166^Ho microbrachytherapy can be an effective neoadjuvant treatment option for canine patients with STS. The resulting tumor volume reduction of 49.0 ± 21.3% facilitated marginal surgical resection of residual tumor and attributed to long survival times, also for relatively large tumors. Our next steps will focus on development of imaging-guided injections and dosimetry to improve safety and efficacy of ^166^Ho microbrachytherapy.

## Data availability statement

The original contributions presented in the study are included in the article/Supplementary material, further inquiries can be directed to the corresponding author.

## Ethics statement

The clinical study was reviewed and approved by the Ethical Committee of the Faculty of Veterinary Medicine, Utrecht University, Utrecht, Netherlands (protocol 2496KGD-holmium-tumoren). Written informed consent was obtained from the owners for the participation of their animals in this study.

## Author contributions

NM and SN drafted, reviewed, and edited the manuscript. JN, GG, JH, and JK reviewed and edited the manuscript. JN, JK, and SN performed the treatments. GG performed histopathological analysis. All authors contributed to the article and approved the submitted manuscript.

## Funding

Writing of the manuscript was part of a large research project for the development of image-guided intratumoral microbrachytherapy of brain tumors using holmium-166 microspheres, which is funded by the Dutch Research Council (Grant number 15499).

## Conflict of interest

Author JN is co-founder and part-time scientific advisor of Quirem Medical which has been acquired by Terumo Europe NV in July 2020. He is entitled to certain milestone payments from Terumo which are related to Quirem's financial, operational, and regulatory performance in the future. Furthermore, he is inventor on the patents related to radioactive microspheres that are assigned to University Medical Center Utrecht Holding BV, Quirem Medical or BASF Corp. The activities of author JN within Quirem Medical are approved and supported by the Board of Directors of the Radboudumc. The remaining authors declare that the research was conducted in the absence of any commercial or financial relationships that could be construed as a potential conflict of interest.

## Publisher's note

All claims expressed in this article are solely those of the authors and do not necessarily represent those of their affiliated organizations, or those of the publisher, the editors and the reviewers. Any product that may be evaluated in this article, or claim that may be made by its manufacturer, is not guaranteed or endorsed by the publisher.
