# Supplementary material for: Intratumoral injection of holmium-166 microspheres as neoadjuvant therapy of soft tissue sarcomas in dogs
Source: Front Vet Sci. 2022 Nov 1;9:1015248. doi: 10.3389/fvets.2022.1015248 (PMC9664058; doi:10.3389/fvets.2022.1015248)
Supplement: Supplementary file 1 [file Data_Sheet_1.docx]

**SUPPLEMENTARY TABLE 1** Computed Tomography (CT; Secura, Philips Medical Systems, Best, Netherlands) protocols and corresponding settings used to image seven canine patients with soft tissue sarcoma before and after holmium-166 microbrachytherapy. The primary used CT protocol varied per patient depending on the location of the tumor. CT of the abdomen and thorax were always acquired to rule out potential locoregional and distant metastases. The values for tube current, exposure time, exposure, and slice thickness are displayed in corresponding order. The tube current varied within each protocol because of differences in animal size and device traction. The pitch was 1.0 for all scans. kVp = Kilovoltage peak, mA = Milliampere, mAs = Milliampere-second.

| **Protocol** | **Tube voltage peak** | **Tube current** | **Exposure time** | **Exposure** | **Slice thickness** | **Soft tissue kernel** |
| --- | --- | --- | --- | --- | --- | --- |
| **Name** | **(kVp)** | **(mA s^-1^)** | **(s)** | **(mAs)** | **(mm)** | **Name** |
| Radius/ulna | 120 | 160-220-260 | 1.0 | 160-220-260 | 2 | PE0 |
| Tarsus | 120 | 200 | 1.0 | 200 | 2 | PE0 |
| Femur | 120 | 160-220 | 0.7-1.0 | 112-220 | 2-5 | PE0-PT0 |
| Pelvis | 120 | 220-240-260 | 1.0-0.7-1.0 | 220-168-260 | 2-5-2 | PE0-AA0-AS0 |
| Abdomen | 120 | 200-240 | 0.7 | 140-168 | 2-3 | PT0-PA0-AA0 |
| Thorax | 120 | 160-200-240 | 0.7 | 112-140-168 | 3-2-2 | PT0 |

**SUPPLEMENTARY TABLE 2** Tumor size measurements and volumes used for tumor response evaluation after holmium-166 microbrachytherapy of seven canine patients with soft tissue sarcoma. For each patient, tumor volume was calculated assuming ellipsoid shape using the three longest perpendicular diameters as measured manually or on CT: Volume = π/6 × length × width × height. *Tumor volume calculated using CT measurements instead of caliper measurements.

| **Patient no.** | **Pre-treatment measurement** | | | | | **Post-treatment measurement** | | | | | **Tumor response** |
| --- | --- | --- | --- | --- | --- | --- | --- | --- | --- | --- | --- |
|  | **Time (days)** | **Length (cm)** | **Width (cm)** | **Height (cm)** | **Tumor volume (cm^3^)** | **Time (days)** | **Length (cm)** | **Width (cm)** | **Height (cm)** | **Tumor volume (cm^3^)** |  |
| **1** | -14 | 3.4 | 4.4 | 5.2 | **40.7** | 5 | 3.0 | 3.4 | 5.2 | **27.8** | -32% |
| **2** | -7 | 2.6 | 4.1 | 2.9 | **16.2** | 29 | 1.5 | 2.3 | 3.2 | **5.8** | -64% |
| **3** | -7 | 5.0 | 6.0 | 7.0 | **110.0** | 36 | 2.0 | 4.2 | 4.5 | **19.8** | -82% |
| **4** | 0 | 5.0 | 5.7 | 3.5 | **52.2** | 84 | 4.7 | 3.4 | 2.9 | **24.3** | -54% |
| **5** | -35 | 4.6 | 7.7 | 8.9 | **165.1** | 21 | 7.0 | 3.0 | 7.0 | **77.0** | -53% |
| **6** | -35 | 9.0 | 6.6 | 8.4 | **261.3*** | 37 | 6.8 | 5.0 | 8.6 | **153.1*** | -41% |
| **7** | -22 | 6.4 | 11.7 | 9.5 | **372.5** | 20 | 6.7 | 11.7 | 7.5 | **307.8** | -17% |

**SUPPLEMENTARY TABLE 3** Radioactivity measurements (MBq) before and after holmium-166 microbrachytherapy of seven canine patients with soft tissue sarcoma. Pre = Pre-treatment measurement, Post = Post-treatment measurement, NA = Not available. Of patient 2, only the amount of injected radioactivity (1048 MBq) was available. For patients 5 and 6, some syringes were measured together after treatment and the total amounts in these syringes are shown (merged cells or *). For patients 4 and 5, the total radioactivity in the vial was lower than the radioactivity in the syringes, likely because part of the radioactivity is shielded by the glass wall from the vial. We did not measure the glass vials again after preparation of the syringes.

| **Patient no.** | **When** | **Vial (MBq)** | **No. of syringes** | **Syringes (MBq)** | **Radioactivity per syringe (MBq)** | | | | | | | | | | | |
| --- | --- | --- | --- | --- | --- | --- | --- | --- | --- | --- | --- | --- | --- | --- | --- | --- |
|  |  |  |  |  | **1** | **2** | **3** | **4** | **5** | **6** | **7** | **8** | **9** | **10** | **11** | **12** |
| **1** | Pre | 1441 | 6 | 1054 | 208 | 196 | 221 | 217 | 146 | 67 |  |  |  |  |  |  |
|  | Post |  |  | 209 | 46 | 26 | 20 | 35 | 30 | 51 |  |  |  |  |  |  |
| **2** | Pre | NA | | | | | | | | | | | | | | |
|  | Post |  |  |  |  |  |  |  |  |  |  |  |  |  |  |  |
| **3** | Pre | 2217 | 12 | 2093 | 82 | 53 | 117 | 82 | 182 | 170 | 187 | 197 | 318 | 225 | 344 | 137 |
|  | Post |  |  | 400 | 25 | 9 | 41 | 16 | 43 | 18 | 17 | 50 | 51 | 47 | 62 | 21 |
| **4** | Pre | 2363 | 11 | 2544 | 267 | 186 | 277 | 155 | 206 | 304 | 151 | 283 | 291 | 287 | 138 |  |
|  | Post |  |  | 252 | 7 | 28 | 44 | 16 | 10 | 26 | 18 | 41 | 31 | 14 | 17 |  |
| **5** | Pre | 2744 | 12 | 2765 | 329 | 319 | 247 | 268 | 199 | 243 | 213 | 207 | 247 | 145 | 255 | 94 |
|  | Post |  |  | 267 | 264 | | | | | | 54* | | | 3 | 54* | |
| **6** | Pre | 2634 | 12 | 2785 | 354 | 293 | 294 | 356 | 231 | 148 | 228 | 202 | 279 | 164 | 182 | 53 |
|  | Post |  |  | 87 | 87 | | | | | | | | | | | |
| **7** | Pre | 3133 | 7 | 2155 | 418 | 357 | 295 | 292 | 306 | 311 | 176 |  |  |  |  |  |
|  | Post |  |  | 344 | 76 | 45 | 37 | 22 | 36 | 72 | 57 |  |  |  |  |  |
